# Life-Threatening Severe Thrombocytopenia and Mild Autoimmune Hemolytic Anemia Associated with Brucellosis

**DOI:** 10.1155/2023/6608279

**Published:** 2023-01-20

**Authors:** Waleed Amsaib M. Ahmed, Khalid Ahmed Khalil, Asma Azwari, Gamal T. A. Ebid, Imran Nazir, Mohamed Hassan Aly

**Affiliations:** ^1^Department of Medicine, Security Forces Hospital, Makkah, Saudi Arabia; ^2^Clinical Pathology Consultant, Security Forces Hospital, Makkah, Saudi Arabia

## Abstract

**Methods:**

We report the case of a 73-year-old Saudi female who presented with severe thrombocytopenia and mild autoimmune hemolytic anemia associated with brucellosis. The coexistence of published cases of two hematological disorders with brucellosis is rare.

**Results:**

Despite the initial treatment with eltrombopag and intravenous immunoglobulin (IVIG), our patient's platelets count remained low and significantly improved after initiation of brucellosis treatment in the form of rifampicin and doxycycline. *Discussion*. We conclude by reviewing the case that in many parts of Saudi Arabia, brucellosis remains a prevalent infection. Hence, it should be considered as a possible diagnosis in febrile individuals with no localizing indications and the presence of severe thrombocytopenia in acute febrile illness. Although it is a rare association, it could be related to brucellosis.

**Conclusion:**

This is our region's first published case of severe thrombocytopenia and mild autoimmune hemolytic anemia associated with brucellosis. It contributes to the literature on the successful use of rifampicin and doxycycline to treat hematological disorders associated with brucellosis.

## 1. Introduction

Brucellosis is a pathogenic disease that affects both domestic and wild animals. It is a serious health issue worldwide, notably in the Mediterranean and the Middle East. The most prevalent *Brucella* species isolated in Saudi Arabia are *B. melitensis* and *B. abortus*; infection from other species has not yet been recorded in the country [1–4]. In Saudi Arabia, males are more affected than females [5]. The true prevalence of brucellosis in humans is uncertain. Human brucellosis has a broad clinical range and offers various diagnostic challenges identical to those seen in multiple illnesses. Coccobacilli of the genus *Brucella* cause the disease, which is transmitted to humans by direct contact with diseased animals and ingesting contaminated animal products [6]. Hematologic disorders such as anemia, leukopenia, and thrombocytopenia linked to brucellosis are rarely reported [7]. *Brucella* can trigger an autoimmune reaction across the body. Two primary causes of thrombocytopenia are decreased platelet synthesis by the bone marrow and increased platelet degradation [8].

On the other hand, autoimmune hemolytic anemia (AIHA) is an uncommon condition characterized by an autoimmune assault on red blood cells. In cases of brucellosis, autoimmune hemolytic anemia is uncommon [9, 10]. Due to molecular mimicry, infection with *Brucella* can cause severe hemolysis and thrombocytopenia [11, 12]. Severe thrombocytopenia is extremely rare as well [13]. Only a few cases of brucellosis-related autoimmune hemolytic anemia and immune thrombocytopenia have been documented in the literature [14, 15]. Antibiotic therapy for human brucellosis aims to mitigate symptoms, control relapses, and decrease complications and death [16].

Therefore, this case report explores the rare association between autoimmune hemolytic anemia and thrombocytopenia with brucellosis in an infected female and defines a practical approach to treating all three manifestations.

## 2. Case Presentation

A 73-year-old female patient presented to the Security Forces Hospital in Makkah, Saudi Arabia, with fever and general body fatigue complaints for two weeks. She also complained of generalized body rashes and one episode of gingival bleeding; however, there was no epistaxis, hematemesis, or melena. Unfortunately, we did not take images of her rash because it was a usual rash with common viral diseases in our area, for example, dengue fever. In addition, the patient had left knee pain for one week before admission. Previous medical track record revealed hypertension which was treated with perindopril and amlodipine. Also, osteoarthritis was reported to be controlled with hydroxychloroquine and celecoxib for the past two years. The patient's family history was unremarkable. There was no history of weight loss and drinking, smoking, or illegal drug usage.

On day 0 of the patient's admittance, physical examination revealed the following vital signs: an initial temperature of 37°C, arterial blood pressure of 110/68 mmHg, heart rate of 94/min, and respiratory rate of 17/min. Anticyclic citrullinated peptides antibody of 0.80 U/ml was reported as normal (0.00–5.00). Antinuclear antibody (ANA) testing was positive, whereas anti-dsDNA antibody 95.4 IU/mL was reported as normal (0.0–200.0). Normal levels of complement C3, 1.03 g/L (normal 0.83–1.93), and complement C4, 0.21 g/L (normal 0.15–0.57), were detected. Both the CXR and ECG were normal. The abdomen was examined with ultrasound and found to be normal. Other physical examinations were ordinary, including a normal heart sound with no murmur or rub, equal air entry bilaterally on chest examination, and normal cranial nerves; power and sensation were intact. Rheumatoid factor, as well as the COVID-19 PCR test, was confirmed as negative. Multiple joint abnormalities exist in both the hands, and there was a left knee effusion with hotness and pain, as well as a skin scar over the right knee from a prior knee replacement. C-reactive protein (CRP) was 19 mg/L, and the erythrocyte sedimentation rate (ESR) was 85 mm/h. Renal and liver function tests were normal, including sodium, potassium, total protein, and albumin levels. She never developed CNS or renal features. Antiviral antibody tests were negative for cytomegalovirus, Epstein-Barr virus, anti-HIV, and hepatitis B and C.

Since the patient has a history of consuming raw milk, ingesting unpasteurized dairy products, and lives in a brucellosis-endemic area (the western region of Saudi Arabia), brucellosis was suspected. Therefore, the blood culture was sent to the microbiology laboratory for further examination.

On day 1, the patient was admitted to the hospital with critically low thrombocytopenia. Platelet counts were only 1 × 10*e*3/*μ*·L with generalized body rash in the form of purpura and petechiae and gum bleeding. The initial impression was idiopathic thrombocytopenic purpura (ITP). The hematological assessment revealed that hemoglobin was 13.5 g/dL, hematocrit was 40.1 percent, and the white blood cell count was 6.4 × 10*e*3/Ul, with an average differential in the initial laboratory tests. The platelets count was 1 × 10*e*3/*μ*·L. Normal Hb and RBCs were confirmed in peripheral blood film assessment. There were some reactive lymphocytes, significant thrombocytopenia, and no aberrant cells. Prothrombin time (PT) is 12.6 seconds, while activated partial thromboplastin time (aPTT) is 36.3 seconds. Initially, the patient received six units of platelets, dexamethasone (40 mg IV daily) for five days, and intravenous immunoglobulin (IVIG) as an urgent intervention for her critically low platelets.

**Figure 1:**
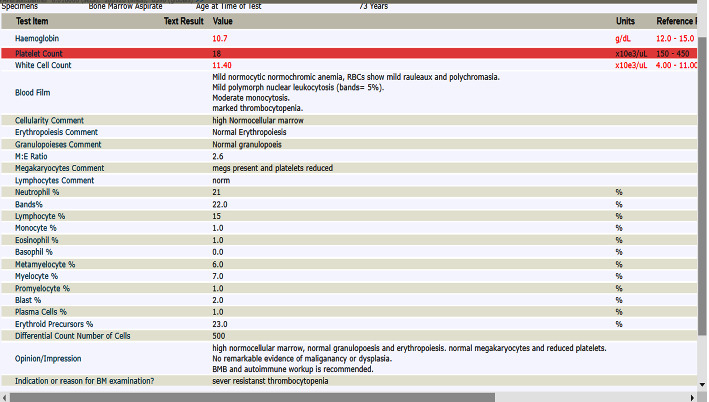
Initial laboratory results for CBC.

On day 7 of admittance to the hospital, the patient's platelets count went up slowly to 18 × 10*e*3/*μ*·L, as shown in Figure 1, so the hematological team decided to start eltrombopag (thrombopoietin receptor agonist) at 50 mg tablet daily.

On day 8 of admission, hemoglobulin started to drop from 13.5 g/dL to 10.6 g/dL; a repeat peripheral blood film showed mild normocytic normochromic anemia; RBCs showed mild rouleaux and polychromasia, mild polymorph nuclear leukocytosis (bands = 5%), moderate monocytosis, and marked thrombocytopenia. A hemolytic anemia workup was sent; haptoglobin was low <0.08 g/L, reticulocytes were high at 2.9% (normal 0–2), and direct antiglobulin was confirmed as positive. Indirect bilirubin was elevated at 15 from a total bilirubin of 23. The patient was diagnosed with autoimmune hemolytic anemia based on a hemolytic workup and severe critically low thrombocytopenia, possible ITP.

On day 10, her platelet count remained low at 12 × 10*e*3/*μ*·L, and her hemoglobin was 9.5. The diagnosis of *Brucella* was made based on the presence of the organism in blood and high *Brucella* serology. The serological analysis showed the *Brucella* antibody titer to be positive (titer, 1 : 2560). Hence, for immediate and effective treatment, the patient was started on doxycycline and rifampicin as a case of brucellosis. At this point, the hematology team decided to stop eltrombopag and IVIG.

The patient underwent bone marrow aspiration on day 10, which revealed high normocellular marrow, normal granulopoiesis and erythropoiesis, normal megakaryocytes, and reduced platelet. No remarkable evidence of malignancy or dysplasia. On day 12 of admission (two days after starting *Brucella* treatment), the platelet count was raised to 42 × 10*e*3/*μ*·L. Three days after *Brucella* treatment (day 13 of admission), the platelet counts improved to 106 × 10*e*3/*μ*·L. After five days of *Brucella* treatment (day 15 of admission), it returned to normal 306 × 10*e*3/*μ*·L. On day 16, the blood culture grew Gram-negative coccobacilli (*Brucella* species).

**Figure 2:**
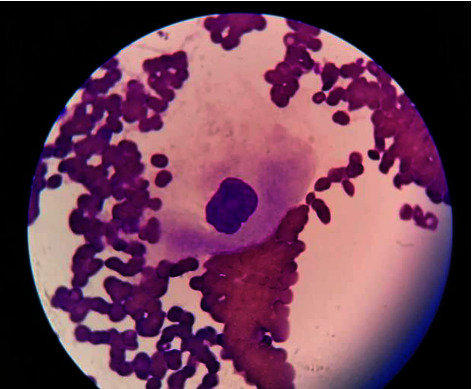
*Brucella*, Gram-negative coccobacilli.

On day 18, after normalization of the platelet count, the patient underwent right knee arthrocentesis, and synovial joint fluid was obtained. Synovial culture grew Gram-negative coccobacilli (*Brucella* species) upon microbial analysis, as shown in Figure 2. The patient was discharged home to be continued on rifampicin and doxycycline. Upon outpatient follow-up five days later, her platelet count remained within the normal range of 301 × 10*e*3/*μ*·L. Her hemoglobin went up to 12.1 g/dL.

## 3. Discussion

In many parts of Saudi Arabia, brucellosis remains a prevalent infection. Here, we have described a case of a 73-year-old female with severe thrombocytopenia and mild autoimmune hemolytic anemia who was infected with brucellosis. It is the first and rare case that a patient with brucellosis has a combination of two hematological disorders. Brucellosis is a deadly and persistent infection that may damage many human organs. Most infections are caused by ingesting unpasteurized dairy products or having occupational contact with infected animals [17]. The incubation period is 1–3 weeks, although it can last for months. *B. melitensis* is globally the most common cause of human brucellosis [17]. It causes acute infection (less than two months). In contrast, other *Brucella* species produce subacute (2–12 months) or chronic (more than one year) infections [18]. In this case, the initial microbial load was examined using blood cultures specifically for brucellemia which came positive. In addition, a blood culture was sent for an invasive bone marrow aspiration procedure. These procedures served as a quantifiable response marker and a relapse prognosis, allowing for appropriate therapy improvements. We discussed with the hematology consultant to do bone marrow biopsy, and the plan was to wait; if patient's platelet count does not improve, then proceed to BM biopsy. Since we diagnosed her with brucellosis and she responded well to *Brucella* treatment, we finally decided not to perform BM biopsy.

*Brucella* spp. is a facultative intracellular pathogen with a unique ability to escape phagocytosis by human macrophages [19]. It is commonly recognized that brucellosis diagnosis is based on clinical symptoms, serology, and bacteriological evidence. The treatment regimen in Saudi Arabia is selected depending on the patient's characteristics and the severity of the condition [20]. Treatment for brucellosis, in this case, is based on a combination of two antibiotics, namely, rifampicin and doxycycline. Eltrombopag was initially used to treat thrombocytopenia. It activates TpoR, stimulating blood platelet production [21]. Eltrombopag was stopped after three days because our hematologist strongly believed that the drug's effect had occurred by that time. Later, the patient was diagnosed with brucellosis, so she was treated with rifampicin and doxycycline, resulting in sustained remission at a 1-monthfollow-up. These antibiotics possess intracellular action (antibiotics penetrate macrophages and are active against the pathogen). These antibiotics helped in decreasing toxicity [22–25]. Reliance on liver function tests is very suggestive, especially at the level of differential diagnosis [26]. Significant differences in hematological and biochemistry markers were found in brucellosis patients studied by Mubaraki [27]. When compared to the healthy group, the activity of the ALT (alanine aminotransferase) enzyme showed a considerable rise in the infected group. In brucellosis patients, albumin levels were significantly lower (34.976–0.702) than in the healthy group [27]. Based on these laboratory findings, which included variations in some hematological inflammatory indices and biochemical markers, we highly suggest using hepatic biomarkers such as AST (aspartate aminotransferase), ALP (alkaline phosphatase), ALT (alanine aminotransferase), and albumin (ALB) in combination with bacteria data, notably in the diagnostic process and follow-up of brucellosis [28]. In our case, rifampicin persists in the infected macrophages' acidic medium and has bactericidal efficacy 48 hours after administration [29]. These findings confirm rifampicin and doxycycline's potential as repurposed remedies for treating brucellosis infection and hematological disorders.

## Data Availability

The data used to support the findings of this study are included within the article.

## Supplementary Materials

Supplementary Materials CARE Guidelines have been followed to write this case; hence, checklist is submitted with the manuscript as a supplementary file in pdf format to the journal.
