# Corrigendum to “Life-Threatening Severe Thrombocytopenia and Mild Autoimmune Hemolytic Anemia Associated with Brucellosis”

**DOI:** 10.1155/2023/9785217

**Published:** 2023-03-08

**Authors:** Waleed Amsaib M. Ahmed, Khalid Ahmed Khalil, Asma Azwari, Gamal T. A. Ebid, Imran Nazir, Mohamed Hassan Aly

**Affiliations:** ^1^Department of Medicine, Security Forces Hospital, Makkah, Saudi Arabia; ^2^Clinical Pathology Consultant, Security Forces Hospital, Makkah, Saudi Arabia

In the article titled “Life-Threatening Severe Thrombocytopenia and Mild Autoimmune Hemolytic Anemia Associated with Brucellosis” [1], some identifiable patient data were unintentionally included. The authors apologize for this error and Figure 1 has been amended to address this. The authors also wish to clarify that written informed consent was obtained from the patient specifically for the publication of this case report.
