# Supplementary material for: Life-Threatening Severe Thrombocytopenia and Mild Autoimmune Hemolytic Anemia Associated with Brucellosis
Source: Case Rep Infect Dis. 2023 Jan 20;2023:6608279. doi: 10.1155/2023/6608279 (PMC9883092; doi:10.1155/2023/6608279)
Supplement: Supplementary Materials — CARE Guidelines have been followed to write this case; hence, checklist is submitted with the manuscript as a supplementary file in pdf format to the journal. [file 6608279.f1.pdf]

CARE Checklist of information

Title **1.** The diagnosis or intervention of primary focus followed by the words “case report” . . .

Life-threatening severe thrombocytopenia and mild autoimmune hemolytic anemia associated with brucellosis: Case Report

Key Words **2.** 2 to 5 key words that identify diagnoses or interventions in this case report, including "case report"

Severe thrombocytopenia, Brucellosis, Autoimmune Hemolytic Anemia, Case Report

Abstract (no references) 3**a.** Introduction: What is unique about this case and what does it add to the scientific literature?

Brucellosis is a pathogenic disease that affects both domestic and wild animals. It is a serious health issue worldwide, notably in the Mediterranean and the Middle East. The most prevalent Brucella species isolated in Saudi Arabia are *B. melitensis* and *B. abortus;* infection from other species has not yet been recorded in the country. In Saudi Arabia, males are more affected than females. The true prevalence of Brucellosis in humans is uncertain. Human Brucellosis has a broad clinical range and offers a variety of diagnostic challenges that are identical to those seen in a variety of other illnesses. Coccobacilli of the genus *Brucella* cause the disease, transmitted to humans by direct contact with diseased animals and the ingestion of contaminated animal products. Hematologic disorders such as anemia, leukopenia, and thrombocytopenia linked to Brucellosis are rarely reported· *Brucella* can trigger an autoimmune reaction across the body. There are two primary causes of thrombocytopenia: decreased platelet synthesis by the bone marrow and increased platelet degradation.

On the other hand, autoimmune hemolytic anemia (AIHA) is an uncommon condition characterized by an autoimmune assault on red blood cells. In cases of Brucellosis, autoimmune hemolytic anemia is uncommon. Infection with *Brucella* can cause severe hemolysis and thrombocytopenia due to molecular mimicry. Severe thrombocytopenia is extremely rare as well. Only a few cases of brucellosis-related autoimmune hemolytic anemia and immune thrombocytopenia have been documented in the literature**.** Antibiotic therapy for human brucellosis aims to mitigate symptoms, control relapses, and decrease complications, sequelae, and death. Therefore, this case report explores the rare association between autoimmune hemolytic anemia and, Thrombocytopenia with Brucellosis in an infected female and defines a practical approach to treating all three manifestations.

Critical thrombocytopenia and autoimmune hemolytic anemia rarely reported in the same patient with brucellosis here we reported a case of critical thrombocytopenia and autoimmune hemolytic anemia associated with brucellosis treated successfully with combination of

Doxycycline and Rifampicin as brucella treatment. Furthermore the mainstay of treatment of these associated hematological disorders is treating the underlying cause (treatment of Brucellosis)

3b. Main symptoms and/or important clinical findings . . . . .

A 73 years old female patient presented to Security Forces Hospital located in Makkah, Saudi Arabia, with fever and general body fatigue complaints for two weeks. She also complained of generalized body rashes and one episode of gingival bleeding; however, there was no epistaxis, hematemesis, or melena. In addition, the patient had left knee pain for one week before admission. Previous medical track record revealed Hypertension which was treated with Perindopril and Amlodipine. Also, osteoarthritis was reported to be controlled with Hydroxychloroquine and celecoxib for the past two years, History of left total knee replacement. The patient's family history was unremarkable. There was no history of weight loss and no drinking, smoking, or illegal drug usage.

Physical exam revealed obese patient,not in distress fully conscious ,oriented ,pale and not jaundiced normal vital signs except for temperature 38.6 ,cardiovascular exam normal first and second heart sounds with no added sound ,Chest exam equal air entry bilateral

Abdomen obese, soft and lax No palpable spleen, liver or any mass

Central nervous system examination entirely intact

Skin bilateral upper and lower limbs petechial rash and few ecchymosis

Right knee swelling was noticed with slight tenderness but no redness or hotness

Left knee examination revealed surgical scar from previous total knee replacement surgery but no other abnormalities.

3c. The main diagnoses, therapeutic interventions, and outcomes.

On Day 0 of the patient's admittance, the following laboratory results were obtained:

Hemoglobin was 13.5 g/dL, Hematocrit was 40.1 percent, and white blood cell count was 6.4x10e3/Ul, with an average differential in the initial laboratory tests. The platelets count was 1 x10e3/u L. Anti-Cyclic Citrullinated Peptides Antibody 0.80 U/ml were reported as normal (0.00 - 5.00), Anti-Nuclear Antibody (ANA)testing was positive, whereas Anti-dsDNA Antibody 95.4 IU/mL was reported as normal (0.0 -200.0). Normal levels of Complement C3, 1.03 g/L (normal 0.83 - 1.93), Complement C4 0.21 g/L (normal 0.15 - 0.57) were detected. Both the CXR and ECG were normal. . Rheumatoid Factor, as well as COVID 19 PCR test, was confirmed as Negative.

C-reactive protein (CRP) was 19 mg/L, and erythrocyte sedimentation rate (ESR) was 85 mm/h. Renal and liver function tests were normal, including sodium, potassium, total protein, and albumin levels. Antiviral antibody tests for cytomegalovirus, Epstein-Barr virus, anti-HIV, and Hepatitis B and C were negative.

Since the patient has a history of consuming raw milk and ingesting unpasteurized dairy products, and she lives in a Brucellosis-endemic area (the western region of Saudi Arabia), Brucellosis was suspected. Therefore, the culture was sent to the microbiology laboratory for further examination.

On Day 1, the patient was admitted to the hospital with critically low thrombocytopenia. Platelets counts were only 1 x10e3/u L with generalized body rash in the form of Purpura and Petechiae and gum bleeding. The initial impression was Idiopathic Thrombocytopenic Purpura (ITP). The hematological assessment revealed that Hemoglobin was 13.5 g/dL, Hematocrit was 40.1 percent, and white blood cell count was 6.4x10e3/Ul, with an average differential in the initial laboratory tests. The platelets count was 1 x10e3/u L. Normal Hb and RBCs were confirmed in peripheral blood film assessment. There were some reactive lymphocytes, significant thrombocytopenia, and no aberrant cells. Prothrombin time (PT) is 12.6 seconds, while activated partial thromboplastin time (aPTT) is 36.3 seconds. Initially patient received six units of platelets and Dexamethasone (40 mg IV daily) for five days, and, Intravenous immunoglobulin (IVIG) as an urgent intervention for her critically low Platelets.

On day 7 of admittance in the hospital, the patient’s Platelets count went up slowly up to 18 x10e3/u L, so the hematological team decided to start Eltromopag (thrombopoietin receptor agonist) at 50 mg tablet daily.

On day 8 of admission, Hemoglobulin started to drop from 13.5 g/dL to 10.6 g/dL; repeated Peripheral blood film showed mild normocytic normochromic anemia, RBCs showed mild rouleaux and polychromasia, and mild polymorph nuclear leukocytosis (bands= 5%), moderate monocytosis, marked thrombocytopenia. A hemolytic anemia workup was sent; haptoglobin was low <0.08 g/L, Reticulocytes were high at 2.9 % (normal 0-2), and Direct antiglobulin was confirmed as Positive. Indirect Bilirubin was high at 15 from total Bilirubin of 23. The patient was diagnosed with Autoimmune Hemolytic Anemia based on hemolytic workup and Severe critically low thrombocytopenia, possible ITP.

On day 10, her platelets count remained low at 12 x10e3/u L, and her hemoglobin was 9.5. The serological analysis showed Brucella antibody titer to be Positive (titer, 1:2560). Hence for immediate and effective treatment, the patient was started on Doxycycline and Rifampicin as a case of brucellosis. At this point, the hematology team decided to stop Eltrombopag and IVIG.

The patient underwent Bone marrow aspiration on Day 10, which revealed high normocellular marrow, normal granulopoiesis, erythropoiesis, normal megakaryocytes, and reduced platelets. No remarkable evidence of malignancy or dysplasia. On day 12 of admission (two days after starting Brucella treatment), The platelets count was raised to 42 x10e3/u L. Three days after Brucella treatment (Day 13 of admission), the platelet counts improved to 106 x10e3/u

L. After five days of Brucella treatment (Day 15 of admission), it returned to normal 306 x10e3/u L. On day 16, the blood culture grew gram Negative coco bacilli (*Brucella* species).

On day 18, after normalization of platelet count, the patient underwent right knee arthrocentesis, and synovial joint fluid was obtained. Synovial culture grew gram-negative coco bacilli (*Brucella* species) upon microbial analysis, as shown in Figures 1, and 2. The patient was discharged home to be continued on Rifampicin and Doxycycline, and upon outpatient follow-up five days later, her platelet count remained within the normal range 301 x10e3/u L. Her Hemoglobin went up to 12.1 g/dL.

3d. Conclusion—What is the main "take-away" lesson(s) from this case?

Critical thrombocytopenia and autoimmune hemolytic anemia could be present in patient with brucellosis although presence of both hematological disorders in the same patient is extremely rare . Furthermore the mainstay of treatment of these hematological disorders associated with brucellosis is the treatment of the underlying cause (treatment of Brucellosis)

Introduction 4. One or two paragraphs summarizing why this case is unique (may include references).

-Our case reported association of 2 hematological disorders in the same patient with brucellosis.

-Critical value of Platelets 1 x10e3/u L was never reported in a patient with Brucellosis

-The Thrombocytopenia corrected with treatment of brucellosis.

Patient Information 5a. De-identified patient specific information. . . . . . Done. . . . .

5b. Primary concerns and symptoms of the patient. . . . .Fever ,gingival bleeding ,skin rash . . . .

5c. Medical, family, and psycho-social history including relevant genetic information . . . . Past Medical History: Hypertension which was treated with Perindopril and Amlodipine. Also, osteoarthritis was reported to be controlled with Hydroxychloroquine and celecoxib for the past two years, History of left total knee replacement. The patient's family history was unremarkable . . . .

5d. Relevant past interventions with outcomes: None

Clinical Findings **6.** Describe significant physical examination (PE) and important clinical findings.

Physical exam revealed obese patient, not in distress fully conscious ,oriented ,pale and not jaundiced normal vital signs except for temperature 38.6 ,cardiovascular exam normal first and second heart sounds with no added sound ,Chest exam equal air entry bilateral

Abdomen obese, soft and lax No palpable spleen, liver or any mass

Central nervous system examination entirely intact

Skin bilateral upper and lower limbs petechial rash and few ecchymosis

Right knee swelling was noticed with slight tenderness but no redness or hotness

Left knee examination revealed surgical scar from previous total knee replacement surgery but no other abnormalities.

Patient was found to have critical thrombocytopenia and autoimmune hemolytic anemia, Brucellosis and brucella related right knee arthritis .

Timeline **7.** Historical and current information from this episode of care organized as a timeline:

On Day 0 of the patient's admittance, the following laboratory results were obtained:

Hemoglobin was 13.5 g/dL, Hematocrit was 40.1 percent, and white blood cell count was 6.4x10e3/Ul, with an average differential in the initial laboratory tests. The platelets count was 1 x10e3/u L. Anti-Cyclic Citrullinated Peptides Antibody 0.80 U/ml were reported as normal (0.00 - 5.00), Anti-Nuclear Antibody (ANA)testing was positive, whereas Anti-dsDNA Antibody 95.4 IU/mL was reported as normal (0.0 -200.0). Normal levels of Complement C3, 1.03 g/L (normal 0.83 - 1.93), Complement C4 0.21 g/L (normal 0.15 - 0.57) were detected. Both the CXR and ECG were normal. . Rheumatoid Factor, as well as COVID 19 PCR test, was confirmed as Negative. C-reactive protein (CRP) was 19 mg/L, and erythrocyte sedimentation rate (ESR) was 85 mm/h. Renal and liver function tests were normal, including sodium, potassium, total protein, and albumin levels. Antiviral antibody tests for cytomegalovirus, Epstein-Barr virus, anti-HIV, and Hepatitis B and C were negative.

Since the patient has a history of consuming raw milk and ingesting unpasteurized dairy products, and she lives in a Brucellosis-endemic area (the western region of Saudi Arabia), Brucellosis was suspected. Therefore, the culture was sent to the microbiology laboratory for further examination.

On Day 1, the patient was admitted to the hospital with critically low thrombocytopenia. Platelets counts were only 1 x10e3/u L with generalized body rash in the form of Purpura and Petechiae and gum bleeding. The initial impression was Idiopathic Thrombocytopenic Purpura (ITP). The

hematological assessment revealed that Hemoglobin was 13.5 g/dL, Hematocrit was 40.1 percent, and white blood cell count was 6.4x10e3/Ul, with an average differential in the initial laboratory tests. The platelets count was 1 x10e3/u L. Normal Hb and RBCs were confirmed in peripheral blood film assessment. There were some reactive lymphocytes, significant thrombocytopenia, and no aberrant cells. Prothrombin time (PT) is 12.6 seconds, while activated partial thromboplastin time (aPTT) is 36.3 seconds. Initially patient received six units of platelets and Dexamethasone (40 mg IV daily) for five days, and, Intravenous immunoglobulin (IVIG) as an urgent intervention for her critically low Platelets.

On day 7 of admittance in the hospital, the patient's Platelets count went up slowly up to 18 x10e3/u L, so the hematological team decided to start Eltromopag (thrombopoietin receptor agonist) at 50 mg tablet daily.

On day 8 of admission, Hemoglobulin started to drop from 13.5 g/dL to 10.6 g/dL; repeated Peripheral blood film showed mild normocytic normochromic anemia, RBCs showed mild rouleaux and polychromasia, and mild polymorph nuclear leukocytosis (bands= 5%), moderate monocytosis, marked thrombocytopenia. A hemolytic anemia workup was sent; haptoglobin was low <0.08 g/L, Reticulocytes were high at 2.9 % (normal 0-2), and Direct antiglobulin was confirmed as Positive. Indirect Bilirubin was high at 15 from total Bilirubin of 23. The patient was diagnosed with Autoimmune Hemolytic Anemia based on hemolytic workup and Severe critically low thrombocytopenia, possible ITP.

On day 10, her platelets count remained low at 12 x10e3/u L, and her hemoglobin was 9.5. The serological analysis showed Brucella antibody titer to be Positive (titer, 1:2560). Hence for immediate and effective treatment, the patient was started on Doxycycline and Rifampicin as a case of brucellosis. At this point, the hematology team decided to stop Eltrombopag and IVIG.

The patient underwent Bone marrow aspiration on Day 10, which revealed high normocellular marrow, normal granulopoiesis, erythropoiesis, normal megakaryocytes, and reduced platelets. No remarkable evidence of malignancy or dysplasia. On day 12 of admission (two days after starting Brucella treatment), The platelets count was raised to 42 x10e3/u L. Three days after Brucella treatment (Day 13 of admission), the platelet counts improved to 106 x10e3/u L. After five days of Brucella treatment (Day 15 of admission), it returned to normal 306 x10e3/u L. On day 16, the blood culture grew gram Negative coco bacilli (*Brucella* species).

On day 18, after normalization of platelet count, the patient underwent right knee arthrocentesis, and synovial joint fluid was obtained. Synovial culture grew gram-negative coco bacilli (*Brucella* species) upon microbial analysis, as shown in Figures 1, and 2. The patient was discharged home to be continued on Rifampicin and Doxycycline, and upon outpatient follow-up five days later, her platelet count remained within the normal range 301 x10e3/u L. Her Hemoglobin went up to 12.1 g/dL.

Diagnostic assessment:

8a. Diagnostic testing (such as PE, laboratory testing, imaging, surveys). . . . .

Hemoglobin was 13.5 g/dL, Hematocrit was 40.1 percent, and white blood cell count was 6.4x10e3/Ul, with an average differential in the initial laboratory tests. The platelets count was 1 x10e3/u L. Anti-Cyclic Citrullinated Peptides Antibody 0.80 U/ml were reported as normal (0.00 - 5.00), Anti-Nuclear Antibody (ANA)testing was positive, whereas Anti-dsDNA Antibody 95.4 IU/mL was reported as normal (0.0 -200.0). Normal levels of Complement C3, 1.03 g/L (normal 0.83 - 1.93), Complement C4 0.21 g/L (normal 0.15 - 0.57) were detected. Both the CXR and ECG were normal. . Rheumatoid Factor, as well as COVID 19 PCR test, was confirmed as Negative. C-reactive protein (CRP) was 19 mg/L, and erythrocyte sedimentation rate (ESR) was 85 mm/h. Renal and liver function tests were normal, including sodium, potassium, total protein, and albumin levels. Antiviral antibody tests for cytomegalovirus, Epstein-Barr virus, anti-HIV, and Hepatitis B and C were negative.

Peripheral blood film showed mild normocytic normochromic anemia, RBCs showed mild rouleaux and polychromasia, and mild polymorph nuclear leukocytosis (bands= 5%), moderate monocytosis, marked thrombocytopenia. A hemolytic anemia workup was sent; haptoglobin was low <0.08 g/L, Reticulocytes were high at 2.9 % (normal 0-2), and Direct antiglobulin was confirmed as Positive. Indirect Bilirubin was high at 15 from total Bilirubin of 23.

The serological analysis showed Brucella antibody titer to be Positive (titer, 1:2560).

Bone marrow aspiration, revealed high normocellular marrow, normal granulopoiesis, erythropoiesis, normal megakaryocytes, and reduced platelets. No remarkable evidence of malignancy or dysplasia.

right knee arthrocentesis, and synovial joint fluid was obtained. Synovial culture grew gram-negative coco bacilli (*Brucella* species)

8b. Diagnostic challenges (such as access to testing, financial, or cultural) None

8c. Diagnosis (including other diagnoses considered) . . . .

critical thrombocytopenia and autoimmune hemolytic anemia associated Brucellosis and brucella related right knee arthritis.

8d. Prognosis (such as staging in oncology) where applicable: Not applicable

Therapeutic interventions:

9a Types of therapeutic intervention (such as pharmacologic, surgical, preventive, self-care) . . . .

9b Administration of therapeutic intervention (such as dosage, strength, duration) . . . .

9c Changes in therapeutic intervention (with rationale)

Initially patient received six units of platelets and Dexamethasone (40 mg IV daily) for five days, and, Intravenous immunoglobulin (IVIG) as an urgent intervention for her critically low Platelets.

When the patient s Platelets count did not improve Eltromopag (thrombopoietin receptor agonist) at 50 mg tablet was added.

When Brucellosis was confirmed we added treatment for it in form of Doxycycline 100 mg orally bid and Rifampicin 600 mg orally daily.

Follow up and Outcome:

10a. Clinician and patient-assessed outcomes (if available) . . .Marked improvement in patient s symptoms and both hematological disorders with Brucellosis treatment

10b. Important follow-up diagnostic and other test results . . . . . .Follow up Platelets returned to normal 306 x10e3/u L and follow up HB improved to 12.1 g/dL.

10c. Intervention adherence and tolerability (How was this assessed?) . . .. patient tolerated the treatment very well.

10d. Adverse and unanticipated events. .None

Discussion:

11a. A scientific discussion of the strengths AND limitations associated with this case report . . . .

11b. Discussion of the relevant medical literature with references. . . . .

11c. The scientific rationale for any conclusions (including assessment of possible causes) . . . .

11d. The primary "take-away" lessons of this case report (without references) in a one-paragraph conclusion

In many parts of Saudi Arabia, Brucellosis remains a prevalent infection. Here, we have described a case of a 73 old female with severe thrombocytopenia and mild autoimmune hemolytic anemia, infected with brucellosis. It is the first and rare case that a patient with brucellosis has a combination of two hematological disorders. *Brucellosis* is a deadly and persistent infection that may damage many human organs. Most infections are caused by ingestion of unpasteurized dairy products or occupational contact with infected animals [1]. The incubation period is 1-3 weeks, although it can last for months. *B. melitensis* is the most common cause of human brucellosis globally [2-3]**.** It causes acute infection (less than two months). In contrast, other *Brucella* species produce subacute (2-12 months) or chronic (more than one year) infections [3]. In this case, the initial microbial load was examined using Blood cultures and an invasive bone marrow aspiration procedure. These procedures served as a quantifiable response marker and a relapse prognosis, allowing for appropriate therapy improvements.

Brucella spp. is a facultative intracellular pathogen with a unique ability to escape phagocytosis by human macrophages [4]. Treatment for brucellosis, in this case, is based on a combination of two antibiotics, namely Rifampicin and Doxycycline. Eltrombopag was initially used to treat thrombocytopenia. It activates TpoR, stimulating blood platelet production [5]. Eltrombopag was stopped after three days because our hematologist strongly believed that the effect of the drug showed has occurred by that time. Later, the patient was diagnosed with brucellosis, so she was treated with Rifampicin and Doxycycline, resulting in sustained remission at a 1-month follow-up. These antibiotics possess intracellular action (antibiotics penetrate macrophages and are active against the pathogen). These antibiotics helped in decreasing toxicity [6-9]. Rifampicin persists in the infected macrophages' acidic medium and has bactericidal efficacy 48 hours after administration [10].

These findings confirm Rifampicin and Doxycycline's potential as repurposed remedies for treating brucellosis infection and hematological disorders.
The strength of our case report is intervention and the outcome were measured and observed during follow up of the patient.
Limitation it is a case report and more case series can confirm our findings.

References:

1. CFSPH. Canine Brucellosis: B. canis. Animal Disease Factsheets [online]. (2007). www.ivis.org.

2. Lucero, N. E., Ayala, S. M., Escobar, G. I., & Jacob, N. R. (2008). Brucella was isolated in humans and animals in Latin America from 1968 to 2006. Epidemiology & Infection, 136(4), 496-503.

3. Seleem, M. N., Boyle, S. M., & Sriranganathan, N. (2010). Brucellosis: a re-emerging zoonosis. Veterinary microbiology, 140(3-4), 392-398.

4. Pappas, G., Akritidis, N., & Tsianos, E. (2005). Effective treatments in the management of brucellosis. Expert opinion on pharmacotherapy, 6(2), 201-209.

5. Lee, H., Lee, J., Hwang, J., Park, S., Kim, N., Kim, K., ... & Jang, S. (2021). Repurposing Eltrombopag for Multidrug Resistant *Staphylococcus aureus* Infections. Antibiotics, 10(11), 1372.

6. Bertrand A. Traitment antibiotique de la brucellose. Presse Med 1994; 23:1128–31.

7. Akova, M. U. R. A. T., Uzun, O. M. R. U. M., Akalin, H. E., Hayran, M., Unal, S. E. R. H. A. T., & Gür, D. (1993). Quinolones in treatment of human brucellosis: a comparative trial of ofloxacin-rifampin versus doxycycline-rifampin. Antimicrobial agents and chemotherapy, 37(9), 1831-1834.

8. Solera, J., Martinez-Alfaro, E., & Espinosa, A. (1997). Recognition and optimum treatment of brucellosis. Drugs, 53(2), 245-256.

9. Zapata, M. R., Herranz, A. G., & Fernandez, J. D. L. M. (1987). Comparative study of two regimens in the treatment of brucellosis. Chemioterapia: international journal of the Mediterranean Society of Chemotherapy, 6(2 Suppl), 360-362.

10. AL-HAJJAJ, M. S., AL-KASSIMI, F. A., AL-MOBEIREEK, A. F., & Alzeer, A. H. (2001). Progressive rise of Mycobacterium tuberculosis resistance to rifampicin and streptomycin in Riyadh, Saudi Arabia. Respirology, 6(4), 317-322.

Patient Perspective **12**. The patient should share their perspective in one to two paragraphs on the treatment(s) they received Satisfied with the outcome

Informed Consent **13**. Did the patient give informed consent? Please provide if requested . . Yes done.
